# A performance evaluation of sthemO 301 coagulation analyzer and associated reagents

**DOI:** 10.1002/jcla.24929

**Published:** 2023-07-10

**Authors:** Adeline Pontis, Mélanie Delanoe, Nathalie Schilliger, Audrey Carlo, Pierre Guéret, Fabienne Nédélec‐Gac, Isabelle Gouin‐Thibault

**Affiliations:** ^1^ Laboratoire d'Hématologie‐Hémostase Centre Hospitalo‐Universitaire de Rennes Pontchaillou Rennes France; ^2^ INSERM, EHESP, Irset (Institut de Recherche en Santé, Environnement et Travail), UMR_S 1085 Université de Rennes Rennes France; ^3^ Diagnostica Stago Asnières‐sur‐Seine France

**Keywords:** analytical performance, analyzer, coagulation, laboratory automation

## Abstract

**Aim:**

The study objective was to evaluate the performance of sthemO 301 system and to compare it with the analyzer used in our university hospital laboratory (STA R Max® 2), for a selection of hemostasis parameters.

**Methods:**

Method comparison (according to CLSI EP09‐A3), carryover (according to CLSI H57‐A), APTT sensitivity to heparin (according to CLSI H47‐A2), HIL level assessment, and productivity were performed using leftover samples from our laboratory (*n* > 1000). Commercial quality control materials were used to evaluate precision (according to CLSI EP15‐A3) and accuracy. The assays tested on sthemO 301 were: PT, APTT (silica and kaolin activators), fibrinogen (Fib), thrombin time (TT), chromogenic and clotting protein C (PC) activity, and von Willebrand factor antigen (VWF:Ag) levels.

**Results:**

All intra‐assay and inter‐assay precision CVs were below the maximal precision limit proposed by the French Group for Hemostasis and Thrombosis (GFHT). Accuracy was verified with bias below GFHT criteria and most Z‐scores were between −2 and +2. No clinically relevant carryover was detected. Silica APTT reagent sensitivity to unfractionated heparin was moderate, as expected. Productivity results were consistent over the 10 repeats performed. The overall agreement between the two systems was excellent for all assays, with Spearman rank correlation coefficient all above 0.9 and slopes of Passing–Bablok correlation near 1 and intercepts close to 0.

**Conclusion:**

For the methods tested, sthemO 301 system met all the criteria to implement a novel coagulation analyzer in the laboratory and result comparability with STA R Max® 2 was good.

## Graphical abstract

Comparison of the performances of sthemO 301 and STA R Max® systems in an university hemostasis laboratory for a selection of hemostasis parameters.

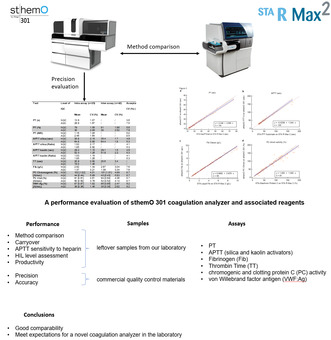

## 1 INTRODUCTION

Coagulation laboratories provide a comprehensive set of clinical tests, with a wide variety of routine and specialized coagulation assays, and expert interpretations for the diagnosis and monitoring of bleeding and thrombotic disorders in hospitalized patients and outpatients. The assays range from routine panels with prothrombin time (PT), activated partial prothrombin time (APTT), and fibrinogen (Fib) to more specific protein activity or antigen measurements.

In order to provide reliable results in an efficient and timely manner, laboratories are often equipped with one or several automated analyzers to perform the assays. With evolving technologies, new instruments are regularly placed on the market, with the aim to improve assay performance and overall efficiency of the coagulation workup in the clinical laboratory.

The sthemO 301 analyzer (Diagnostica Stago, France) belongs to this new generation of fully automated hemostasis analyzers. It is designed for the qualitative and/or quantitative in vitro determination of coagulation parameters by clotting (chronometric), chromogenic, or immunological methods in citrated plasma samples. The dedicated reagents and test procedures developed by the manufacturer for use on this new analyzer enable, among others, improved traceability (with unique reagent vial ID) and optimized performance through adapted pipetting and mixing sequences. In order to increase the system throughput while decreasing the technician's hands‐on time, the overall instrument workflow management for samples, disposables, reagents loading and unloading and for the measurement pace was improved, with an unique measuring cell and higher reagent vial capacity. The aim of the present study was to evaluate and publish for the first time, the performance of sthemO 301 system and to compare it with the analyzer used in our university hospital laboratory (STA R Max® 2), for a selection of hemostasis parameters.

## 2 MATERIALS AND METHODS

### 2.1 Study design

This study was performed in the hematology laboratory of the University Hospital of Rennes, France. All citrated plasma samples tested (from blood collected into 0.109 mol/L sodium citrate tubes), anonymized for the study, were leftovers from patients' samples received in the laboratory between January and March 2022 after completion of all testing, as waived by the Ethics Committee of our institution. When appropriate, samples were selected according to their results obtained in routine as described hereafter, and after selection all samples were anonymized, with no option to get back to patients' identities. Samples were analyzed on the STA R Max® 2 and the sthemO 301 analyzers (Diagnostica Stago, Asnières‐sur‐Seine, France) with the corresponding reagents for PT, APTT (silica and kaolin activators), Fib, thrombin time (TT), chromogenic and clotting protein C (PC) activity, anti‐Xa levels (only on STA R Max® 2), and von Willebrand factor antigen (VWF:Ag) levels.

The following reagents, with their corresponding calibrators and controls (Diagnostica Stago), were used: STA‐NeoPTimal® 10, STA‐PTT Automate® 5, STA‐C.K. PREST® 5, STA‐Liquid Fib®, STA‐Thrombin® 10, STA‐Stachrom® Protein C, STA‐Staclot® Protein C 1, STA‐Liquid Anti‐Xa®, STA‐Liatest® VWF:Ag on the STA R Max®; and sthemO PT M, sthemO PTT A (silica), sthemO CK Prest (kaolin), sthemO Fib M, sthemO Thrombin M, sthemO PC Clot M, sthemO PC Chrom and sthemO VWF:Ag, on the sthemO 301 analyzer, using locally determined reference times for sthemO PTT A and sthemO CK Prest. Concerning PT, the ISI and reference time provided by the manufacturer were used for INR calculation.

Protein C and VWF:Ag assays were calibrated on each analyzer with their respective calibrators provided by the manufacturer. As per package insert information, the calibrators assigned levels were determined against their respective secondary standard of corresponding International Standards for these parameters, namely 02/342 established in 2006 for protein C and 07/316 established in 2009 for VWF:Ag. This calibration led to results expression for these parameters in % on the instrument but are here reported in both % and in IU/mL.

PT, APTT, Fib, anti‐Xa, and TT were performed on fresh plasma samples only. PC and VWF:Ag were performed on frozen plasma samples: plasmas were frozen after double centrifugation at −20°C for less than a week. Frozen plasmas were thawed at 37°C for 5 min just before testing.

### 2.2 Accuracy

Twenty‐three external quality assessment samples from our regular EQA program (Qualiris QC Premium S1‐2021, Qualiris QC Premium S2‐2021, Diagnostica Stago) were tested on sthemO 301 and compared with the mean values of our regular peer group using bias and the Z‐score metrics. Acceptable bias was set according to the French Group on Hemostasis and Thrombosis (GFHT) recommendations.^1^

### 2.3 Intra‐assay and inter‐assay precision

Intra‐assay precision was assessed according to CLSI EP15 protocol.^3^ In short, two different quality control plasma samples were tested five times per run on five different runs (5 non‐consecutive days) to obtain 25 replicates (*n* = 25) on sthemO 301.

Inter‐assay precision was tested through the repeated measurement of internal QC (IQC) samples, being measured twice a day on 21 different days.

Precision results were compared to the acceptable CV determined by GFHT.^1^

### 2.4 Carryover

Inter‐sample carryover was assessed according to CLSI H57‐A protocol^4^ for sthemO PT, sthemO PTT A, sthemO CK Prest and sthemO Fib reagents using two pools of fresh plasma samples. The two pools consisted of: normal plasma samples i.e within the reference range (pool A) and pathologic plasma samples i.e outside the reference range levels (pool B). Each pool was then divided into three parts (A1–A3 and B1–B3).

To assess carryover from normal samples to pathological samples, the test sequence was A1–A2–A3–B1–B2–B3, and for carryover from pathological samples to the normal samples, the test sequence was B1–B2–B3–A1–A2–A3. All test sequences were repeated 10 times.

Inter‐reagent carryover was tested using five aliquots of a normal pool of plasma. Each aliquot was measured with sthemO PTT A, sthemO Fib, and sthemO PTT A again, and this test series on the five samples was replicated five times.

### 2.5 Method comparison studies

Method comparison studies were performed with samples covering, as much as possible, the whole measuring range of each assay with the aim of testing around 100 samples for each parameter as recommended by CLSI EP09‐A3.^5^

Samples were selected for this study according to the initial result of their coagulation tests, and were then tested again in parallel on sthemO 301 and STA R Max® 2 to avoid any bias in results due to delay in testing. Method comparison was run on the following assays: PT, APTT (both silica and kaolin activators), TT, Fib, PC (chromogenic and clotting activities), and VWF:Ag.

### 2.6 Sensitivity of APTT assays to unfractionated heparin

To estimate the sensitivity to heparin of the two sthemO APTT reagents available, 20 samples from patients receiving unfractionated heparin (UFH), covering as much as possible the range between 0.10 and 0.70 anti‐Xa IU/mL, were tested (APTT clotting times on sthemO 301 analyzer, anti‐Xa levels on STA R Max® 2). From this, we attempted to determine the APTT values corresponding to UFH anti‐Xa therapeutic range (0.30–0.70 IU/mL).

Finally, as per CLSI H47‐A2^6^ recommendations, results were plotted on a graph APTT = f(anti‐Xa), to assess the sthemO APTT reagents overall sensitivity to heparin.

### 2.7 HIL

Hemolysis, icterus, and lipemia/turbidity (HIL) indices are often measured on serum and plasma to validate sample quality, especially since sample's color may influence coagulation test results, though for clotting assays, mechanical clot detection is known to be more robust than optical detection.^7^, ^8^ The ability to detect presence of HIL was evaluated by checking the consistency of levels provided by the sthemO 301 preanalytical module, in comparison to a visual check by the operator and to the HIL indices reported through the Expert Preanalytical Check module of STA R Max® 2. For this, we used 20 hemolyzed samples, 10 icteric samples. and three lipemic samples. In addition, we tested two samples with both obvious hemolysis and icterus.

### 2.8 Productivity

We assessed laboratory productivity with sthemO 301 instrument using 50 plasma samples from our routine activity tested for PT, APTT, and Fib. We measured the duration of sample loading, reagent loading, the time needed by the instrument to give the first and the whole set of results, and the time to unload the samples. This experiment was repeated on 10 independent occasions and the average throughput was then determined.

### 2.9 Statistical analysis

Statistical analyses were done with MedCalc software version 17.4.4 and Microsoft Excel using version Microsoft 365 MSO version (16.0.14326.20850).

According to CLSI H57‐A, inter‐sample carryover was assessed using a two‐tailed Student's *t* test to compare the mean value of sample B1 with that of sample B3 (for normal to pathological carryover), and the mean value of sample A1 with that of sample A3 (for pathological to normal carryover). Similarly, inter‐reagent carryover was tested comparing the mean APTT values obtained in the first rank of testing, to the mean APTT values obtained after fibrinogen measurement, using a two‐tailed Student's *t* test. In both cases, a *p*‐value below 0.05 was considered statistically significant.

As proposed by CLSI EP09‐A3, to test method agreement and consistency, we used Passing–Bablok regression and a Bland–Altman plots. The Spearman rank correlation coefficient was also used to further evaluate the strength and direction of the correlation. Values of the mean bias on the Bland–Altman graphs were compared to the limits proposed by GFHT for the corresponding parameters.^1^

## 3 RESULTS

### 3.1 Accuracy

We were able to evaluate accuracy on a total of 23 EQA samples from previous campaigns. We compared the results to our peer‐group reports. Table 1 summarizes the data from these experiments.

**TABLE 1:** Accuracy evaluation on sthemO 301 analyzer in comparison to peer methodologies.

Assay	Range of value	Minimal bias	Maximal bias	Average bias	Acceptable bias^1^
PT (s)	<17	−6.25%	−4.08%	−5.22%	±11.9%
	[17–23]	−10.40%	−5.14%	−6.81%	±19.5%
	≥23	−8.75%	−2.96%	−5.53%	±38.7%
PT (%)	0–29	−4.49%	0.56%	−2.80%	±27.7%
	>29	−9.20%	1.83%	−4.67%	±10.0%
PT (INR)	<1.6	−1.53%	6.36%	3.75%	±7.0%
	[1.9–3.0]	−3.45%	2.29%	−0.68%	±13.4%
	≥3.0	−6.42%	−3.02%	−4.27%	±23.4%
APTT silica (s)	[33–50]	−3.22%	2.71%	−0.24%	±21.0%
	[50–69]	−7.07%	2.71%	−0.24%	±17.7%
	≥69	−10.67%	−5.49%	−7.31%	±17.9%
APTT silica (ratio)	All levels	−14.95%	1.01%	−5.42%	±18.7%
TT (s)	All levels	−7.56%	2.57%	−5.03%	NA
Fib (g/L)	<1.6	6.67%	20.39%	9.76%	±38.9%
	[1.5–2[	6.67%	20.39%	10.44%	±19.8%
	≥2.3	1.66%	5.04%	2.98%	±14.8%
PC chromogenic (%) [IU/mL]	<98 <0.98	−5.54%	8.70%	0.66%	±23.3%
VWF:Ag (% or IU/mL)	All levels	−12.24%	−1.19%	−6.42%	±17.9%

For each EQA sample tested and each test methodology, the observed bias to peers was below the acceptable bias proposed by GFHT.^1^

Of note, for one sample the Z‐score indicated the need for a corrective action for several tests^2^: APTT (in seconds and ratio), Fib, and VWF:Ag. However, as other samples were tested during the same run with the same reagents and with Z‐score values between −2 and +2, we concluded that the observed discrepancy result was likely due to reconstitution error.

### 3.2 Intra‐assay and inter‐assay precision

Intra‐assay and inter‐assay precisions results are shown in Table 2.

**TABLE 2:** Intra‐ and inter‐assay precision on sthemO 301 analyzer.

Test	Level of IQC	Intra‐assay (*n* = 25)		Inter‐assay (*n* = 42)		
		Mean	CV (%)	Mean	CV (%)	Acceptable CV (%)^1^
PT (s)	NQC	13.9	1.07	/	/	3.6
	AQC	29.9	1.67	/	/	7.5
PT (%)	NQC	79	1.55	81	1.86	5.6
	AQC	30	2.09	30	2.52	7.9
PT (INR)	NQC	1.16	1.05	/	/	5.0
	AQC	2.46	1.64	/	/	8.5
APTT silica (s)	NQC	35.8	2.15	35.4	1.7	4.9
	AQC	64.8	0.86	64.4	1.8	5.3
APTT silica (Ratio)	NQC	1.02	2.17	/	/	4.1
	AQC	1.85	0.92	/	/	5.3
APTT kaolin (s)	NQC	29.2	1.15	29.1	1.5	4.9
	AQC	52.4	1.26	51.9	2.3	5.3
APTT kaolin (Ratio)	NQC	1.03	1.15	/	/	4.1
	AQC	1.85	1.26	/	/	5.3
TT (s)	NQC	20.9	2.58	20.6	3.4	/
	AQC	19.0	1.46	/	/	/
Fib (g/L)	NQC	2.92	1.34	2.97	3.04	7.6
	AQC	1.37	3.21	1.39	3.34	7.6
PC chromogenic (%) [IU/mL]	NQC	102 [1.02]	4.01	101 [1.01]	4.89	8.7
	AQC	44 [0.44]	5.89	43 [0.43]	5.83	8.7
PC Clot (%) [IU/mL]	NQC	91 [0.91]	4.61	94 [0.94]	4.36	8.7
	AQC	50 [0.50]	3.76	52 [0.52]	4.14	8.7
VWF:Ag (%) [IU/mL]	NQC	87 [0.87]	1.82	86 [0.86]	2.47	9.2
	AQC	36 [0.36]	1.46	35 [0.35]	2.62	9.2

Abbreviations: AQC, abnormal quality control; APTT, activated partial thromboplastin time; CV, coefficient of variation; Fib, Fibrinogen; NQC, normal quality control; PC, protein C, PT, prothrombin time; TT, thrombin time; VWF: Ag, von Willebrand Factor antigen.

Intra‐assay CVs ranged between 0.86% and 5.89%, for all parameters.

Inter‐assay CVs ranged from 1.5% to 5.5%, which is always lower than the GFHT acceptable limits.^1^

### 3.3 Carryover

No statistically significant inter‐sample carryover was observed (all *p*‐values > 0.05) except:

- From normal to abnormal sample on PT assay in seconds only (*p*‐value = 0.02), the abnormal PT moving from 34.5 to 34.8 s.
- From abnormal to normal sample on APTT assay with sthemO CK Prest (*p*‐value = 0.01), the normal APTT moving from 27.5 to 27.8 s.

When considering the actual differences, the impact is a difference of ±0.3 seconds for both cases.

The statistical relevance of such a low difference is consistent with assay precision results as the observed average difference is higher than the standard deviation of the assay.

No statistically significant inter‐reagent carryover was observed either.

### 3.4 Method comparison between STA R® Max 2 and sthemO 301 analyzers

Sample size, minimal and maximal observed values, and Passing–Bablok regression results for all assays are presented in Table 3. All Spearman rank correlation coefficients are above 0.95 (except for TT) and we notice that regression slopes are close to 1 and intercepts close to 0. Bland–Altman plots enable to see that average biases observed in these method comparison studies are all below the maximal acceptable bias proposed by GFHT.^1^

**TABLE 3:** Method comparison data: number of samples, minimal (min), and maximal (max) values observed on STA R Max® 2 and sthemO 301 analyzers, and Spearman rank correlation coefficient and Passing–Bablok regression fit parameters.

Test	Number of samples	Min value (STA R Max® 2 – sthemO 301)	Max value (STA R Max® 2 – sthemO 301)	Spearman rank correlation coefficient (*r*)	Intercept [95% CI]	Slope [95% CI]
PT (s)	100	12.0–10.9	75.9–71.7	0.974	−0.44 [−1.01; −0.09]	0.96 [0.93; 0.99]
PT (%)	100	11–11	117–114	0.975	0.85 [−0.59; 2.47]	0.96 [0.94; 0.98]
PT (INR)	100	0.91–0.92	6.17–5.60	0.971	0.07 [0.02; 0.11]	0.95 [0.91; 0.98]
APTT silica (s)	100	26.0–26.0	170.7–163.8	0.950	−0.03[−1.54; 1.26]	1.04 [1.01; 1.09]
APTT silica (ratio)	100	0.77–0.77	5.02–4.87	0.951	−0.003 [− 0.047; 0.044]	1.05 [1.02; 1.10]
APTT kaolin (s)	99	22.4–22.0	89.6–79.5	0.986	1.11 [0.34; 2.01]	0.96 [0.93; 0.99]
APTT kaolin (ratio)	99	0.66–0.78	2.64–2.80	0.985	0.05 [0.02; 0.08]	1.14 [1.10; 1.18]
TT (s)	44	15.0–14.0	59.0–61.2	0.919	2.93 [−1.00; 4.41]	0.75 [0.66; 1.00]
Fib (g/L)	95	0.48–0.49	9.93–9.16	0.998	0.07 [0.04; 0.10]	0.98 [0.97; 0.99]
PC chromogenic (%)	81	8–6	197.0–187.0	0.974	1.00 [−1.00; 3.58]	1.00 [0.97; 1.04]
PC chromogenic (IU/mL)	81	0.08–0.06	1.97–1.87	0.974	0.01 [−0.01; 0.04]	1.00 [0.97; 1.04]
PC clot (%)	73	5–5	188.0–191.0	0.981	−0.73 [−2.87; 0.44]	0.98 [0.94; 1.01]
PC clot (IU/mL)	73	0.05–0.05	1.88–1.91	0.981	−0.007 [−0.029; 0.004]	0.98 [0.94; 1.01]
VWF:Ag (%)	99	8–4	439–420	0.997	−3.36 [−4.54; −2.27]	1.04 [1.02; 1.06]
VWF:Ag (IU/mL)	99	0.08–0.04	4.39–4.20	0.997	−0.03 [−0.05; −0.02]	1.04 [1.02; 1.06]

Abbreviation: CI, confidence interval.

The method comparison on TT shows a tendency to shorter clotting times for TT on sthemO 301 analyzer with sthemO Thrombin, in comparison to STA R Max®2 with STA‐Thrombin®, and a weaker correlation coefficient (0.919). However, only 44 samples could be used for this analysis, as many samples selected for the study eventually had clotting times longer than the upper limit of the assay (>60 s on STA R Max® 2; >80 s on sthemO 301).

Besides, for INR, there were some differences mostly in the highest INR values, however, those differences were all below 15%.

**FIGURE 1:**
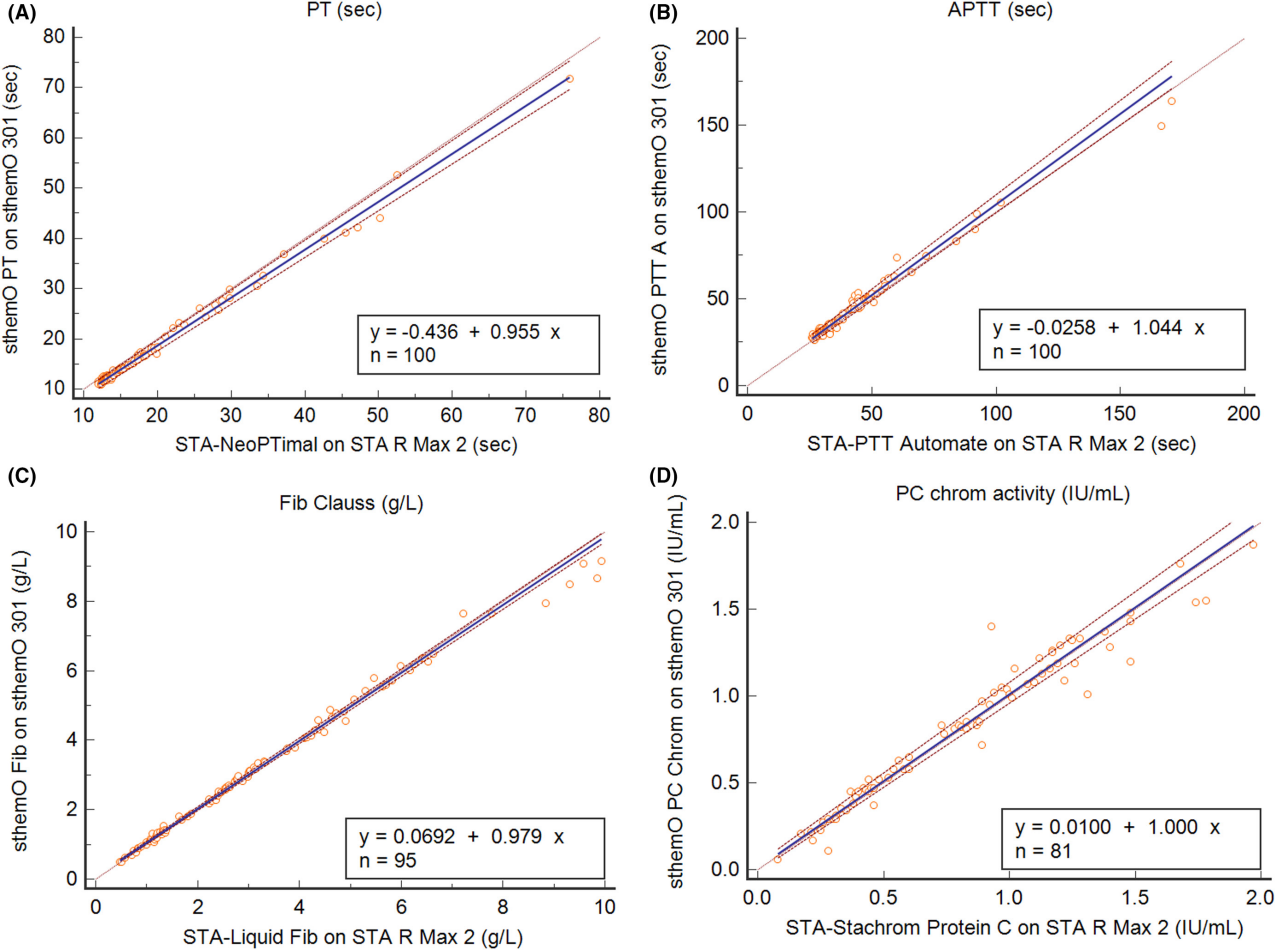
Correlation between values measured on sthemO 301 and STA R Max® 2 for different assays: (A) PT in seconds (B), APTT in seconds, (C) fibrinogen level in g/L (Clauss method), and (D) PC chromogenic activity in IU/mL.

**FIGURE 2:**
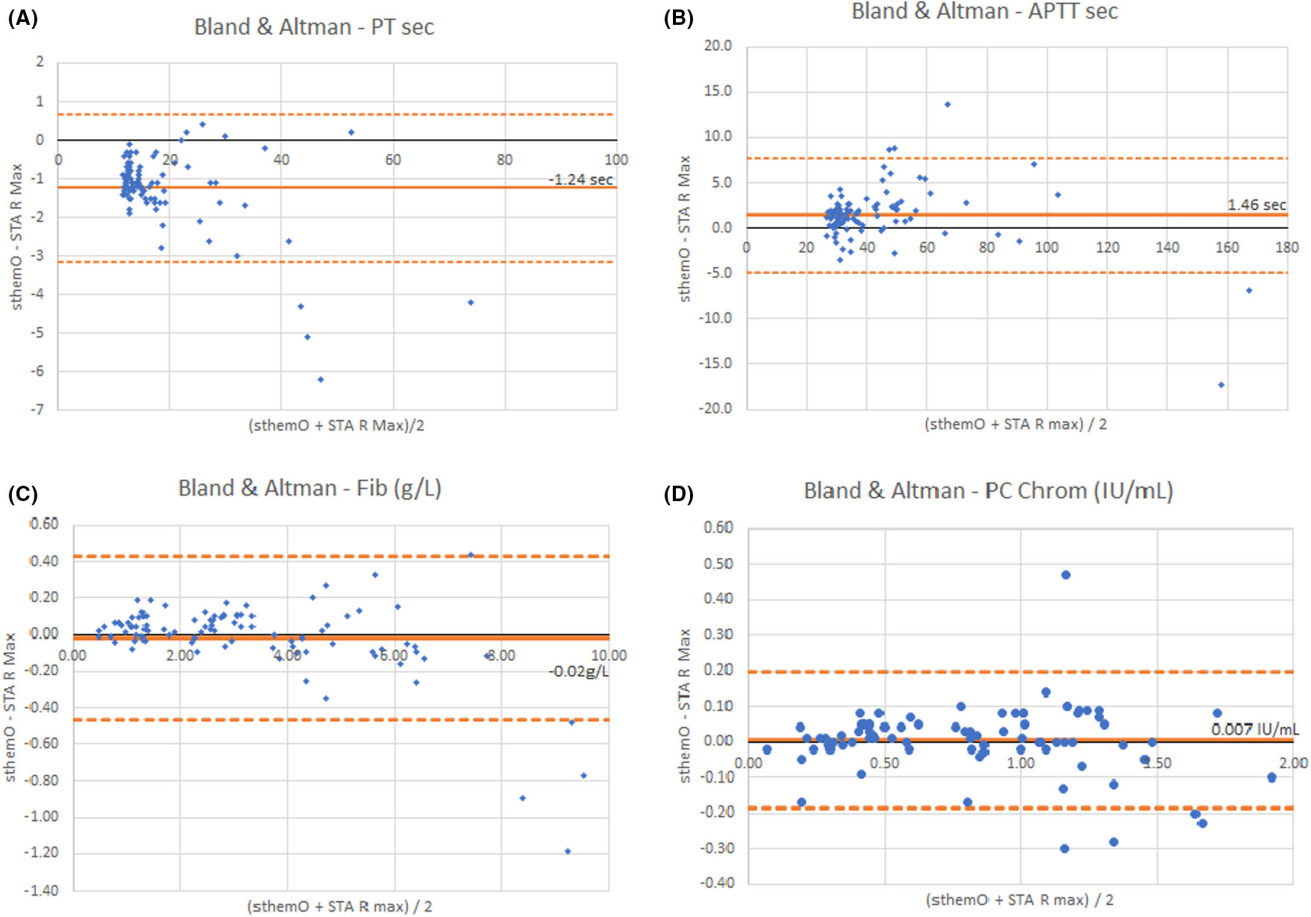
Bland and Altman plots for sthemO 301 versus STA R Max® 2. (A) PT in seconds, (B) APTT in seconds, (C) fibrinogen level in g/L (Clauss method), and (D) PC chromogenic activity in IU/mL.

As graphical examples, the method comparison for PT (in seconds), APTT (in seconds), PC chromogenic, and fibrinogen assays are presented in Figures 1 and 2.

### 3.5 Sensitivity of APTT assay to heparin

**FIGURE 3:**
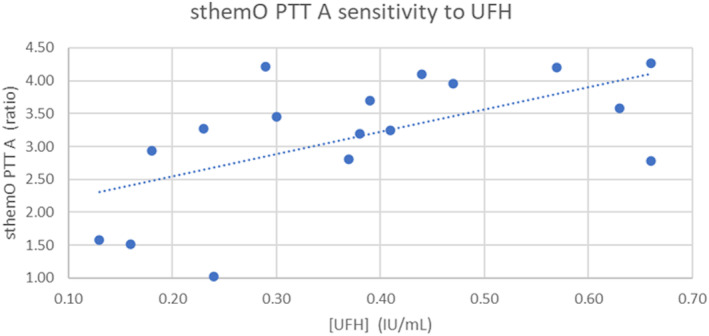
Sensitivity of sthemO PTT A to UFH: correlation between APTT clotting time ratios and UFH anti‐Xa levels.

The correlation between sthemO PTT A values and anti‐Xa levels measured on STA R Max® was low as shown in the scatter plot (Figure 3). The APTT values corresponding to UFH anti‐Xa therapeutic range of 0.30 to 0.70 IU/mL assessed using the 20 samples are 101 to 148 in seconds, or 2.90 to 4.20 in ratio.

As all samples but one had an APTT ratio >1.5 it was not possible to determine the exact sensitivity to UFH of sthemO PTT A.

### 3.6 HIL

The comparison of HIL indices determined by STA R Max® 2 and sthemO 301 was consistent overall as observed on 20 samples with visible hemolysis, 10 samples with icterus three visible lipemia, and two samples with combined hemolysis and icterus. Only three icteric samples had slightly lower index levels on STA R Max® 2 than on sthemO 301 for high index levels. All indices were also globally consistent with visual inspection, and the small discrepancies mentioned earlier were in favor of sthemO 301 determination.

### 3.7 Productivity

The sample sets used for the different runs of throughput, consisted on average in each run of 49% of samples within the normal reference ranges for PT, APTT, and Fib and 51% of samples falling outside the normal reference ranges for the same assays.

The average throughput observed over 10 days to run PT, APTT, and Fib on 50 samples each time was 34 minutes (ranging from 33 to 36 min). It took on average 1 min 30 to load the samples and 2 min to load the reagents. Time to first result was 4 min on average (minimum = 3 min and maximum = 5 min).

## 4 DISCUSSION

In this study, we aimed at assessing and publishing for the first time the performance of the new sthemO 301 analyzer in comparison with the STA R Max® 2. The study included comparison of clotting (chronometric), chromogenic, and immunological methods using sthemO 301 analyzer dedicated reagents, calibrators, and controls. Overall, the performance and agreement between the two analyzers were excellent, meeting the validation criteria, and our results enable us to better anticipate the performance that can be achieved with sthemO 301 system in a hemostasis laboratory.

Indeed, our tests on EQA samples confirmed the accuracy of PT, APTT, TT, Fib, PC clotting, PC chromogenic, and VWF:Ag assays on sthemO 301. Besides, the intra‐ and inter‐assay precisions were all within the French acceptable limits.^1^ No significant carryover was demonstrated except for PT in seconds from normal to abnormal samples and for kaolin‐based APTT from abnormal to normal samples. Nevertheless, the extent of the observed absolute differences in these experiments (±0.3 s) is not clinically relevant for these assays and the statistical observation is most likely due to the very high precision of the device, leading to conclude statistically significant such small differences.

In the method comparison studies, all results showed great consistency between sthemO 301 and STA R Max® 2 systems. TT values, measured on a more limited number of samples than for other reagents and parameters, were slightly shorter on sthemO 301 analyzer. The two thrombin time reagents of the two systems contain the same thrombin amount but could be slightly different by other aspects not disclosed by the manufacturer. This constant bias on TT with sthemO 301 could be addressed using appropriately and locally defined reference ranges. There were also few differences in the highest INR values, all below 15% and therefore without any clinical relevance.^9^ It is important to note that the reagents used for PT on the two analyzers were different.

Unfortunately, sthemO anti‐Xa reagent was still on development during the study, so no anti‐Xa reagent could be tested on this analyzer and the relationship between sthemO PTT A values and anti‐Xa levels was analyzed only using STA‐Liquid anti‐Xa® values on STA R Max® 2. This is a limitation to the evaluation of sthemO PTT A sensitivity to heparin, that would deserve further investigation when anti‐Xa measurement will be available on sthemO analyzer. However, this approach reflects the reality of a number of laboratories where anti‐Xa levels are not accessible or done on another coagulation platform than APTT measurement. The relationship between sthemO PTT A clotting times and anti‐Xa levels was low and this was expected due to the low specificity of APTT, especially in critical ill patients.^10^ We were able to calculate the sthemO PTT A clotting time values corresponding to anti‐Xa activities of 0.30 and 0.70 IU/mL. Even if this range needs to be validated on a larger number of samples, it shows the sensitivity of this silica‐based APTT to UFH, a sensitivity which is known to be highly variable among reagents.^11^ Detection of HIL was comparable between analyzers. Evaluation of productivity was limited because, due to organizational constraints in our laboratory, we could not compare the sthemO 301 throughput to that of our STA R Max® 2. Nonetheless, we observed very consistent throughput on the sthemO 301 analyzer over the 10 different runs we performed.

One last limitation of our evaluation is the lack of access to normal donor samples, to establish or at least verify locally reference intervals for the different assays. This would have to be done if the sthemO 301 system was to be implemented for routine use in any coagulation clinical laboratory.

In conclusion, for the methods tested, we found a good comparability between the new sthemO 301 analyzer and the STA R Max® 2, using their respective dedicated reagents. Overall, the results we obtained with sthemO 301 meet the expectations one can have while implementing a novel coagulation analyzer in the laboratory.

## CONFLICT OF INTEREST STATEMENT

AC is full‐time employee of Diagnostica Stago. The study was financially supported by Diagnostica Stago.

## Data Availability

The data that support the findings for this study are available from the corresponding author upon reasonable request.
